# Imprisonment and mortality among adults with neurodevelopmental disabilities in New South Wales, Australia, 2001–2015: a data-linkage cohort study

**DOI:** 10.1136/bmjopen-2025-102805

**Published:** 2025-10-13

**Authors:** Erin Spike, Preeyaporn Srasuebkul, Azar Kariminia, Tony Butler, Julian Trollor

**Affiliations:** 1 Kirby Institute, Faculty of Medicine and Health, University of New South Wales, Sydney, New South Wales, Australia; 2 The National Centre for Excellence in Intellectual Disability Health, Faculty of Medicine and Health, University of New South Wales, Sydney, New South Wales, Australia; 3 School of Population Health, Faculty of Medicine and Health, University of New South Wales, Sydney, New South Wales, Australia

**Keywords:** Prisons, Mortality, Disabled Persons, Developmental neurology & neurodisability

## Abstract

**Abstract:**

**Objectives:**

(1) Examine the associations between imprisonment history and mortality among adults with neurodevelopmental disabilities and (2) examine the associations between receipt of disability services and post-release mortality among adults with neurodevelopmental disabilities released from prison.

**Design:**

Population-based data-linkage cohort study using historical administrative data.

**Setting:**

New South Wales (NSW), Australia.

**Participants:**

67 217 adults aged ≥18 years (59.1% male) with one or more neurodevelopmental disabilities in NSW, Australia, from July 2001 to June 2015.

**Outcome measures:**

The main outcome measure was all-cause mortality. In the full cohort, we used Cox regression to examine the associations between release from imprisonment and all-cause mortality. In a subcohort of those released from prison, we used Poisson regression to examine the associations between receipt of disability services and post-release all-cause mortality.

**Results:**

3.3% of participants (n=2214) were imprisoned and released at least once during follow-up. In all age groups<55 years, age-specific all-cause mortality rates were higher in those released from prison than in those who were not imprisoned. In Cox regression models, after adjusting for sociodemographic factors, release from prison was associated with higher all-cause mortality compared with never imprisonment (adjusted HR (aHR) 1.50 (95% CI 1.17 to 1.92)); however, this association was no longer present after further adjustment for mental illness, substance use and physical comorbidity (aHR 1.02 (95% CI 0.79 to 1.31)). Among those released from prison, being known to the prison disability service was associated with higher post-release mortality (adjusted mortality rate ratio (aMRR) 1.97 (95% CI 1.18 to 3.30)), whereas receipt of community disability services was not associated with post-release mortality (aMRR 1.09 (95% CI 0.55 to 2.15)).

**Conclusions:**

Among adults with neurodevelopmental disabilities, mortality was increased among those released from prison compared with their peers who had not been imprisoned, although this was largely explained by health-related factors, including mental illness, substance use and physical comorbidity. Comprehensive policy and service system responses are required to meet the health and safety needs of people with neurodevelopmental disabilities who have complex needs, including criminal legal system involvement, mental illness and substance use.

**STRENGTHS AND LIMITATIONS OF THIS STUDY**

- Large, population-based study using multiple administrative data sources to ascertain neurodevelopmental disabilities.
- Possibility of selection bias due to underidentification of neurodevelopmental disabilities in administrative datasets.
- Use of health and prison datasets for cohort selection may have led to over-representation of people with mental or physical health conditions or experience of imprisonment.
- Possibility of some outcome, covariate or exposure misclassification due to linkage or clerical error; likely to be non-differential.
- Results are not necessarily generalisable to other jurisdictions due to the diversity of legal, health and disability service systems.

## Introduction

 Neurodevelopmental disabilities refer to the differences in brain processing and consequent functional impairments associated with a range of childhood-onset conditions.^1^ These include intellectual disability, autism, attention-deficit hyperactivity disorder (ADHD) and fetal alcohol spectrum disorder (FASD). Previous studies indicate that neurodevelopmental disabilities, including intellectual disability,^2^ ADHD^3^ and FASD,^4^ are markedly over-represented among people in prison. This raises concerns about the criminalisation of disability and contributing factors, including socioeconomic marginalisation, inadequate health and disability supports, and ableist discrimination,^5^ as well as impacts of imprisonment on service provision and health outcomes in this population.

Both neurodevelopmental disabilities and imprisonment are associated with substantial health inequities, of which mortality is one indicator. People with neurodevelopmental disabilities are at an increased risk of early death^6^,^9^ and disproportionately experience mental illness,^10^,^13^ substance use,^14 15^ accidental injury^16 17^ and physical conditions such as cerebral palsy, sensory impairments and epilepsy.^18^,^21^ Likewise, mental illness and substance use disorders are common in prison populations,^22^ as are adverse social determinants of health, including low educational attainment, childhood adversity, financial deprivation and homelessness.^23^ Risks of hospitalisation^24^ and death^25^ are elevated after prison release, with high rates of fatal^26^ and non-fatal^27^ drug overdose in the first fortnight. However, there is little evidence regarding the extent to which imprisonment is associated with health outcomes—including mortality—among people with neurodevelopmental disability. This is important given the distinct needs, service interactions and patterns of marginalisation experienced by this population. Existing evidence suggests that imprisonment is associated with an increased prevalence of mental illness and substance use within cohorts of people with neurodevelopmental disabilities,^28 29^ mirroring associations in the general population.

Various factors may contribute to harm among people with neurodevelopmental disabilities during and after imprisonment. In Australia, inadequate disability recognition, supports and accommodations have been noted throughout criminal legal systems, including prisons,^30^,^33^ with a lack of culturally appropriate disability services for First Nations people,^34^ who are incarcerated at 15 times the rate of other New South Wales (NSW) adults.^35^ Within prisons, people with neurodevelopmental disabilities face increased risks of mentally and/or physically traumatic experiences such as segregated custody, restraint, bullying and assault,^36 37^ and healthcare access is often disrupted and below community standards.^38^ After release, barriers to accessing health and disability services and poor transitional support and planning are common, exacerbating the challenges of re-entry.^39^ People with additional needs such as mental illness, substance use and homelessness—often termed ‘complex support needs’—are particularly marginalised and poorly served by fragmented health, disability and welfare services.^40^ While receiving disability services post-release reduces reincarceration,^41^ research concerning its association with health outcomes is minimal.

We are aware of only one study examining mortality in people with neurodevelopmental disabilities following imprisonment,^42^ which found that all-cause mortality was similar in people with and without intellectual disability released from prison. We aimed to extend the limited literature on imprisonment and health in people with neurodevelopmental disabilities by examining the associations between imprisonment and mortality among adults with neurodevelopmental disabilities in NSW. We also aimed to identify whether receipt of disability support services in prison or post-release was associated with post-release mortality.

## Methods

### Data sources and linkage

We performed a data-linkage cohort study using historical data from multiple NSW administrative datasets. Dataset commencement and end dates differed, but most had complete coverage for the study period of 1 July 2001–31 June 2015. First, we used five disability service datasets: the NSW Department of Ageing, Disability and Home Care Disability Services Minimum Dataset (DS-MDS), recording state-funded disability service provision (July 2005–June 2016); the NSW Ombudsman dataset, recording reviewable residential care deaths (December 2002–December 2015); the NSW Public Guardian dataset, recording Public Guardian involvement in decision-making (January 1994–June 2016); the NSW Disability Programs in Public Schools dataset, recording disability service provision within public schools (January 2011–June 2015); and the Corrective Services NSW (CSNSW) Statewide Disability Services (SDS) dataset, recording disability service provision within adult prisons (May 1987–November 2015). Second, we used three NSW Health datasets: the Emergency Department Data Collection (EDDC), recording emergency department presentations to public hospitals (January 2005–June 2016); the Admitted Patient Data Collection (APDC), recording public and private hospital admissions (July 2001–June 2016); and the Mental Health Ambulatory Data Collection (MH-AMB), recording public community mental health service provision (January 2001–December 2015). Third, we used the CSNSW Offender Integrated Management System (OIMS), recording imprisonments in adult prisons (January 1994–May 2016); the NSW Registry of Births, Deaths and Marriages Death Registrations dataset (RBDM-DR), recording deaths occurring in NSW (January 1994–March 2016); and the Australian Co-ordinating Registry’s Cause of Death Unit Record File (COD-URF), recording deaths and causes of deaths in NSW (January 1985–December 2013).

The NSW Health Centre for Health Record Linkage (CHeReL) performed probabilistic linkage between datasets with Choicemaker software, using names, birthdates and addresses. The linkage method uses probability weighting supplemented by manual review of intermediate-probability matches, with estimated false-positive and false-negative rates of 0.5%, and undergoes regular quality assurance checks.^43^ The CheReL assigned a unique number to each individual and removed identifying information before supplying data.

### Context and setting

NSW has the largest population (7.6 million in 2015)^44^ and the largest adult prison population of any Australian state/territory (11 797 on 30 June 2015).^45^ Prison disability services are provided by Statewide Disability Services, a division of Corrective Services NSW. From 2013 to 2020, community disability services underwent a staged transition to the federally funded National Disability Insurance Scheme (NDIS), which uses a personalised market-based funding model to purchase services from the private sector.^46^ However, throughout the study period (ie, until 2015 inclusive), the vast majority of disability services in NSW were provided or procured by the NSW state government under a block-funding model.

### Study population

Our study population comprised all adults with intellectual disability or autism recorded in any disability or health dataset or any other neurodevelopmental disability recorded in the APDC or MH-AMB, who were alive and aged ≥18 years at any time between 1 July 2001 and 30 June 2015. Participants entered observation on the later of 1 July 2001 or their 18th birthday, with follow-up until 30 June 2015 or death. As described previously,^7 47^ we identified intellectual disability and autism using dataset-specific codes recorded in the disability datasets or diagnoses recorded in the APDC, MH-AMB and EDDC. We identified other neurodevelopmental disabilities using the following International Classification of Diseases 10th Revision—Australian Modification (ICD-10-AM) codes recorded in the APDC or MH-AMB (primary or secondary diagnoses): specific speech, language, learning and motor disorders (F80-F83); Rett syndrome (F84.2); other/unspecified developmental disorders (F88-F89); hyperkinetic disorders (F90); tic disorders (F95) and stuttering and cluttering (F98.5-F98.6). Since neurodevelopmental disabilities have a childhood onset by definition and typically involve lifelong impairment, we assumed that the disability commenced before the 18th birthday even if first identified in a dataset after this age. We also identified a subcohort of those released from an adult prison at least once during the study period (the prison release subcohort).

**Figure 1:**
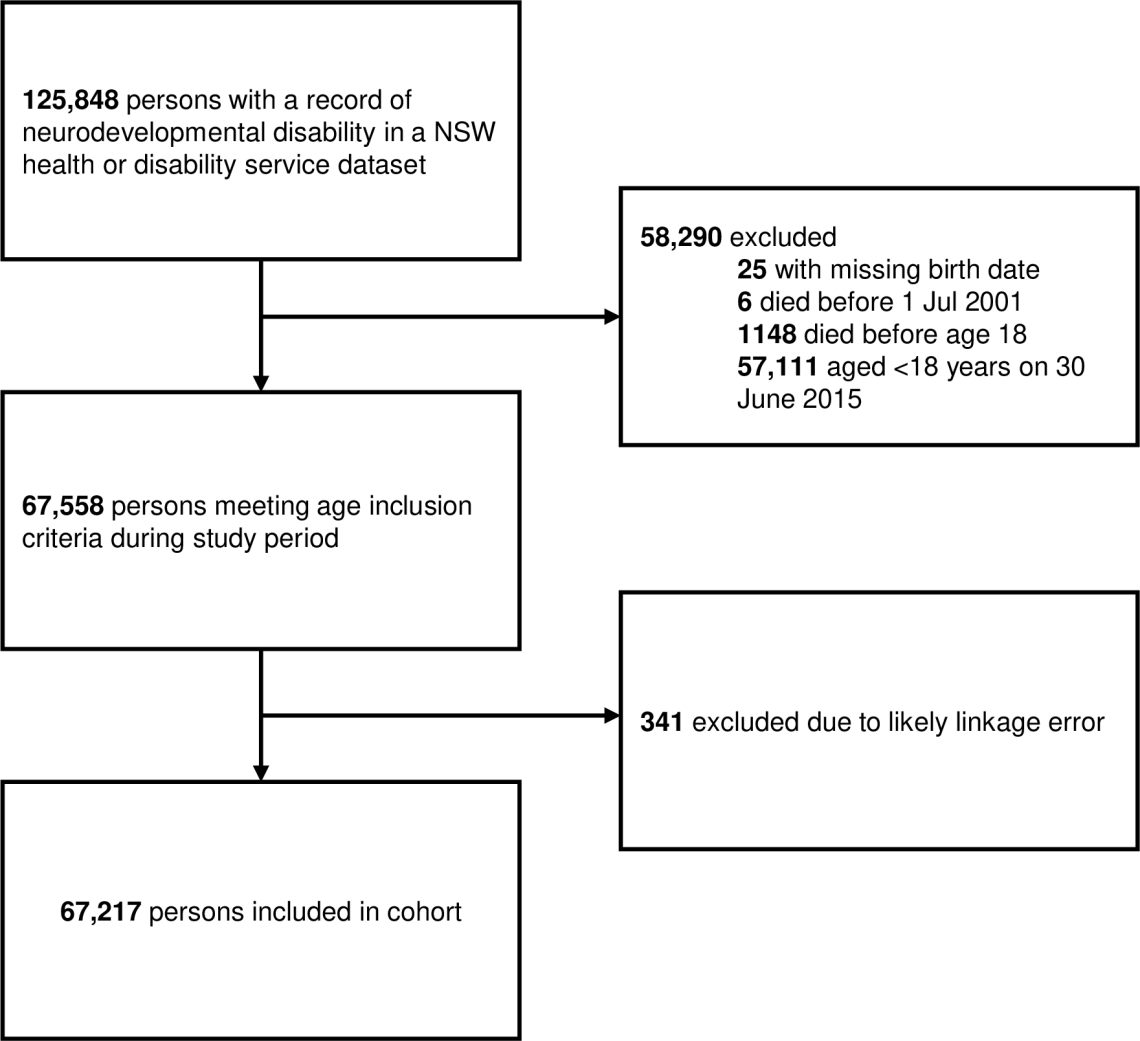
Cohort selection flowchart. NSW, New South Wales

We excluded persons with suspected linkage error based on comparisons between birth/death dates and event dates within datasets (figure 1; detailed criteria in online supplemental material).

### Outcomes

The primary outcome was all-cause mortality, ascertained from the RBDM and COD-URF. Using the underlying cause of death in the COD-URF, we also identified cause-specific deaths due to external causes (substance use disorders (ICD-10: F10-F19), abuse of non-dependence-producing substances (F55) or external causes of morbidity and mortality (Chapter XX)), which were further subcategorised into unintentional drug-induced deaths (ICD-10: F11-F16, F18-F19, F55, X40-X44) and suicide, including undetermined intent (X60-X84, Y10-Y34, Y87.0, Y87.2); and disease-related causes (all causes other than external and undefined).

### Main exposure

The main exposure was imprisonment history, that is, any imprisonment (remand or sentenced) and subsequent release from a NSW adult prison during the study period, ascertained from imprisonment start and end dates recorded in OIMS. This was treated as a time-updated variable (never imprisoned/in prison/post-release). As mortality is lower in prison versus community settings,^48^ and due to few in-prison deaths in our cohort (n<10), time in prison was excluded from follow-up. As our interest was in adult imprisonments, we excluded imprisonments wherein a person was aged <18 years for the entirety.

### Sociodemographic, health-related and disability-related variables

We derived sociodemographic characteristics from the DS-MDS, APDC and EDDC. Characteristics treated as fixed were sex; Aboriginal and/or Torres Strait Islander identity; residential Index of Relative Socioeconomic Disadvantage (IRSD), an area-based socioeconomic disadvantage measure obtained by mapping residential Statistical Area Level 2 (SA2) to IRSD quintiles published by the Australian Bureau of Statistics^49^; and residential remoteness, obtained by mapping residential SA2 to Australian Statistical Geography Standard remoteness areas.^50^ Age was time-updated and categorised into bands (18–24 years, 25–34 years, 35–44 years, 45–54 years, 55–64 years, ≥65 years).

We measured co-occurring mental illness, drug-related harm and alcohol-related harm during the study period as time-updated binary variables (ever/never present). We defined co-occurring mental illness as any diagnosis in Chapter V (Mental and Behavioural Disorders) of the ICD-10-AM recorded in the APDC or MH-AMB (primary/secondary diagnoses), excluding codes used for cohort selection and substance use-related codes (F10-F19). We ascertained alcohol-related harm (ICD-10-AM: F10, R78.0, T51, X45, X65, Y15, Y90-91) and drug-related harm (ie, harms related to drugs of addictive potential; ICD-10-AM: F11-F16, F18-F19, R78.1-R78.4, T40, T41.2, T42.2-T42.3, T42.4, T43.6, T52-T53, T59.0, T59.8, X41-X42, X46, X61-X62, X66, Y11-Y12, Y16) using diagnoses (primary and secondary) in the APDC, MH-AMB and EDDC. As some EDDC diagnoses were coded using the International Classification of Diseases 9th Revision (ICD-9) or the Systematized Nomenclature of Medical Terminology—Australian release (SNOMED-CT-AU), we mapped these ICD-10-AM codes to ICD-9 and SNOMED-CT-AU codes. We measured physical comorbidities using the updated Charlson Comorbidity Index (CCI),^51^ a validated score predicting mortality risk in a given year based on inpatient diagnoses in the previous year (possible range 0–24; categorised into 0 and ≥1). This was time-updated each birthday using APDC diagnoses (primary and secondary) in the previous year of age.

We measured mental illness, drug-related and alcohol-related hospitalisations during the study period as binary variables (ever/never hospitalised), defined as any acute hospital admission in the APDC with a primary diagnosis of mental illness, drug-related harm or alcohol-related harm (using the above ICD-10-AM codes). We excluded planned, single-day admissions. We measured community mental health service use (ever/never used) based on clinical contacts recorded in the MH-AMB and receipt of state-funded community disability services (ever/never received) recorded in the DS-MDS.

In the prison-release subcohort, we measured whether a person was known to the prison disability service at the time of release (a proxy for receipt of disability services in prison), based on whether they had previously appeared as a client in the SDS dataset. We also measured receipt of state-funded community disability services at the time of release using the DS-MDS. As the DS-MDS aggregated this information by financial year, disability services were assumed to be present at release if they were recorded in the same financial year. If multiple releases occurred, these variables were time-updated at each release.

### Statistical analysis

We calculated descriptive characteristics stratified by imprisonment history during follow-up and, for the prison release subcohort, by survival status at the end of follow-up. Ethics approvals did not permit reporting on Aboriginal and/or Torres Strait Islander identity.

For the whole cohort, we calculated age-specific all-cause mortality rates per 1000 person-years stratified by imprisonment history. For deaths with cause of death information available (n=4546), we calculated proportions in each cause of death category, stratified by imprisonment history. We used Cox regression to model univariable and adjusted associations between imprisonment history and all-cause mortality. Model 1 adjusted for sociodemographic covariates (age, sex, Aboriginal and/or Torres Strait Islander identity, residential IRSD, residential remoteness). Model 2 further adjusted for health-related covariates (co-occurring mental illness, drug-related harm, alcohol-related harm, CCI score), which we modelled as time-updated covariates to capture changing morbidity patterns given the youth of our cohort. All covariates were selected as potential confounders a priori. Due to few deaths (n<10) among persons aged ≥55 years with a prison release history, we limited regression analyses to persons aged <55 years (n=61 431). We verified the proportional hazards assumption for each model graphically using Kaplan-Meier, log-log and Schoenfeld residual plots.

In the prison release subcohort, we calculated mortality rates per 1000 person-years in the following time periods post-release: 1 month, 2–3 months, 4–6 months, 7–12 months and ≥12 months (taking 30 days per month). To examine the associations between receipt of disability services (during imprisonment and post-release) and post-release all-cause mortality, we modelled mortality rate ratios (MRRs) using random-effects Poisson regression (taking post-release person-years as the exposure time and adjusting standard errors for within-participant clustering to account for multiple releases). We also limited these analyses to persons aged <55 years, and for post-release disability services, to releases on/after 1 July 2005 (as post-release disability service data were only available from this date). Given few post-release deaths (n=79), we adjusted these analyses for limited sociodemographic covariates only (time-updated continuous age in years; sex; Aboriginal and/or Torres Strait Islander identity).

We used SAS 9.4 and STATA 18 for all analyses. We followed the Strengthening the Reporting of Observational Studies in Epidemiology reporting guidelines.^52^

### Patient and public involvement

There was no direct patient or public involvement in this study. We gratefully acknowledge the individuals whose anonymised records provided data.

## Results

### Descriptive statistics

Our cohort comprised 67 217 participants with 676 094 total person-years of follow-up (median 13.5 years per person; IQR 5.8–14.1 years). 59.1% (n=39 696) were male, and most (54.8%, n=36 854) were aged 18–24 years at entry (table 1). 83.0% (n=55 780) had an intellectual disability, 14.6% (n=9820) were autistic and 23.5% (n=15 825) had another neurodevelopmental disability.

**Table 1:** Descriptive characteristics of adults with neurodevelopmental disabilities in New South Wales 2001–2015 by imprisonment status during follow-up*

	Not imprisoned during follow-up (n=64 908)		Imprisoned during follow-up (n=2309)		Total (n=67 217)	
	n	%	n	%	n	%
Sex, n (%)						
Male	37 603	57.9	2093	90.6	39 696	59.1
Female	27 245	42.0	216	9.4	27 461	40.9
Missing	60	0.1	0	0.0	60	0.1
Age, n (%)						
18–24 years	35 284	54.4	1570	68.0	36 854	54.8
25–34 years	9221	14.2	474	20.5	9695	14.4
35–44 years	8451	13.0	192	8.3	8643	12.9
45–54 years	6189	9.5	56	2.4	6245	9.3
55–64 years	3195	4.9	17	0.7	3212	4.8
≥65 years	2568	4.0	0	0.0	2568	3.8
Index of Relative Socioeconomic Disadvantage, n (%)						
Most disadvantaged quintile	12 919	19.9	696	30.1	13 615	20.3
Second-most disadvantaged quintile	12 161	18.7	526	22.8	12 687	18.9
Medium disadvantaged quintile	13 468	20.7	523	22.7	13 991	20.8
Second-least disadvantaged quintile	12 503	19.3	312	13.5	12 815	19.1
Least advantaged quintile	10 605	16.3	191	8.3	10 796	16.1
Missing	3252	5.0	61	2.6	3313	4.9
Remoteness area, n (%)						
Major cities	41 957	64.6	1383	59.9	43 340	64.5
Inner regional	14 656	22.6	611	26.5	15 267	22.7
Outer regional, remote and very remote	6101	9.4	268	11.6	6369	9.5
Missing	2194	3.4	47	2.0	2241	3.3
Neurodevelopmental disability type, n (%)						
Intellectual disability	53 706	82.7	2074	89.8	55 780	83.0
Autism	9735	15.0	85	3.7	9820	14.6
Other neurodevelopmental disability	15 191	23.4	634	27.5	15 825	23.5
Ever co-occurring mental illness, n (%)^†^	20 338	31.3	1766	76.5	22 104	32.9
Ever drug-related harm, n (%)^†^	6199	9.6	1434	62.1	7633	11.4
Ever alcohol-related harm, n (%)^†^	5042	7.8	1086	47.0	6128	9.1
Ever mental illness hospitalisation, n (%)^†^	10 285	15.8	1047	45.3	11 332	16.9
Ever drug-related hospitalisation, n (%)^†^	2603	4.0	753	32.6	3356	5.0
Ever alcohol-related hospitalisation, n (%)^†^ ^†^	1586	2.4	444	19.2	2030	3.0
Ever attended community mental health service, n (%)^†^	16 332	25.2	2006	86.9	18 338	27.3
Ever received community disability services, n (%)^†‡^	37 665	58.0	772	33.4	38 437	57.2
Charlson Comorbidity Index score, n (%)						
0	64 610	99.5	2299	99.6	66 909	99.5
≥1	298	0.5	10	0.4	308	0.5
Died during follow-up, n (%)^†^	5353	8.2	86	3.7	5439	8.1

* For sex, Index of Relative Socioeconomic Disadvantage and remoteness area, missing values were combined with most common category for all analyses (ie, ‘male’, ‘medium disadvantaged’ quintile and ‘major cities’, respectively). No other variables had missing values.

† Measured at the end of follow-up. All other variables measured at baseline.

‡ Denominator includes 941 individuals who died on/before 1 July 2005 (the commencement date for community disability service data).

3.4% (n=2309) of the cohort were imprisoned during follow-up, and 2214 had at least one release (9995 releases total; median 3 per person, IQR 1–6). Compared with those not imprisoned, those imprisoned had higher proportions of co-occurring mental illness (76.5% in those imprisoned vs 31.3% in those not imprisoned), mental illness hospitalisation (45.3% vs 15.8%), community mental health service use (86.9% vs 25.2%), drug-related harm (62.1% vs 9.6%), drug-related hospitalisation (32.6% vs 4.0%), alcohol-related harm (47.0% vs 7.8%) and alcohol-related hospitalisation (19.2% vs 2.4%). A lower proportion of those imprisoned received state-funded disability services during follow-up compared with those not imprisoned (33.4% vs 58.0%).

Among the prison-release subcohort (table 2), drug-related and alcohol-related harm and drug-related and alcohol-related hospitalisations were more prevalent among those who died during follow-up than those who survived, whereas co-occurring mental illness was less prevalent. Those who died were older than those who survived and a greater proportion had multiple imprisonments (88.2% vs 77.4%).

**Table 2:** Descriptive characteristics of adults with neurodevelopmental disabilities in New South Wales released from prison from 2001 to 2015 by survival status at end of follow-up

	Alive at the end of follow-up (n=2129)		Died during follow-up (n=85)		Total (n=2214)	
	n	%	n	%	n	%
Male, n (%)*	1929	90.6	75	88.2	2004	90.5
Age, n (%)						
18–24 years	1189	55.8	29	34.1	1218	55.0
25–34 years	556	26.1	27	31.8	583	26.3
35–44 years	267	12.5	19	22.4	286	12.9
45–54 years	NR	–	NR	–	93	4.2
55–64 years	NR	–	NR	–	29	1.3
≥65 years	NR	–	NR	–	5	0.2
Index of Relative Socioeconomic Disadvantage, n (%)*						
Most disadvantaged quintile	641	30.1	20	23.5	661	29.9
Second-most disadvantaged quintile	496	23.3	15	17.6	511	23.1
Medium disadvantaged quintile	532	25.0	27	31.8	559	25.2
Second-most advantaged quintile	286	13.4	13	15.3	299	13.5
Most advantaged quintile	174	8.2	10	11.8	184	8.3
Remoteness area, n (%)*						
Major cities	1309	61.5	58	68.2	1367	61.7
Inner regional	565	26.5	22	25.9	587	26.5
Outer regional, remote and very remote	255	12.0	5	5.9	260	11.7
Neurodevelopmental disability type, n (%)						
Intellectual disability	1916	90.0	77	90.6	1993	90.0
Autism	NR	–	NR	–	81	3.7
Other neurodevelopmental disability	586	27.5	22	25.9	608	27.5
Ever co-occurring mental illness, n (%)†	1644	77.2	59	69.4	1703	76.9
Ever drug-related harm, n (%)†	1331	62.5	63	74.1	1394	63.0
Ever alcohol-related harm, n (%)†	1007	47.3	56	65.9	1063	48.0
Ever hospitalised for mental illness, n (%)†	977	45.9	37	43.5	1014	45.8
Ever hospitalised for drug use, n (%)†	703	33.0	33	38.8	736	33.2
Ever hospitalised for alcohol use, n (%)†	401	18.8	32	37.6	433	19.6
Ever attended community mental health service, n (%)†	1856	87.2	70	82.4	1926	87.0
Ever received community disability services, n (%)^†‡^	722	33.9	16	18.8	738	33.3
Charlson Comorbidity Index score, n (%)						
0	NR	–	NR	–	2170	98.0
≥1	NR	–	NR	–	44	2.0
Number of imprisonments^†^						
1	482	22.6	10	11.8	492	22.2
2–3	648	30.4	26	30.6	674	30.4
4–8	728	34.2	38	44.7	766	34.6
≥9	271	12.7	11	12.9	282	12.7
Client of prison disability service at any prison release						
Yes	1312	61.6	58	68.2	1370	61.9
Received community disability services at any prison release^†§^						
Yes	522	26.2	11	15.3	533	25.9

* There were no missing values for sex in the prison release subcohort. Number and proportion of missing values for Index of Relative Socioeconomic Disadvantage and remoteness area not reported due to cell counts <5 or to prevent back-calculation of cells with count <5.

† Measured at the end of follow-up. All other variables measured at first release during follow-up.

‡ Denominator includes n<5 individuals who died on/before 1 July 2005 (the 17 commencement date for community disability service data).

§ Restricted to persons who had at least one release on/after 1 July 2005 (n=2063).

NR, not reported due to cell count <5 or to prevent back-calculation of cells with count <5.

### Mortality rates by imprisonment history

**Figure 2:**
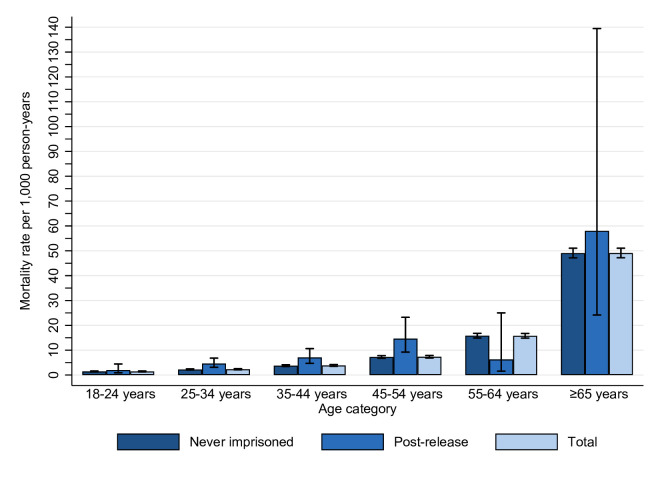
Age-specific all-cause mortality rates by imprisonment history among adults with neurodevelopmental disability in New South Wales, 2001–2015 (n=67 217). Excludes deaths occurring in prison (n<10). Excludes data from persons (n<5) with all follow-up time spent in prison.

Figure 2 illustrates age-specific all-cause mortality rates by imprisonment history. In the 25–34-year, 35–44-year and 45–54-year age groups, mortality rates were higher in those post-release compared with those never imprisoned. This was also the case for those aged 18–24 years, although 95% CIs for the estimates overlapped. In the 55–64-year age group, mortality rates were lower in those post-release than those not imprisoned, while mortality rates in those aged ≥65 years were similar. Because few people aged ≥55 years were released from prison, there were few deaths and person-years of exposure in these age groups, resulting in wide CIs.

### Cause of death by imprisonment history

Table 3 presents cause of death by imprisonment history during follow-up for those aged <55 years at death; results for those ≥55 years are not reported due to small numbers of releases and post-release deaths. External and undefined causes, largely unintentional drug-induced and suicide deaths, accounted for 53.7% of deaths in those released from prison during follow-up, vs 14.5% in those not imprisoned.

**Table 3:** Cause of death by imprisonment history during follow-up among adults with neurodevelopmental disability aged <55 years at death in New South Wales, 2001–2015*

	Not imprisoned during follow-up		Released from prison during follow-up		Total	
	n	%	n	%	n	%
Disease-related	1287	85.5	25	46.3	1312	84.2
Suicide	63	4.2	10	18.5	73	4.7
Unintentional drug-induced	54	3.6	14	25.9	68	4.4
All external†	218	14.5	29	53.7	247	15.8
Total	1505	100.0	54	100.0	1559	100.0

Data source: Cause of death unit record file held by the New South Wales Ministry of Health Secure Analytics for Population Health Research and Intelligence.

* Only includes deaths for which cause of death information was available. Excludes deaths that occurred in prison (n<10). Data for members of cohort aged ≥55 years at death not shown due to multiple cells with counts <5.

† Includes undefined deaths (overall n<20; exact number not displayed to prevent cell counts <5)

### Associations between imprisonment history and all-cause mortality

Table 4 presents HRs for associations between imprisonment history and all-cause mortality. In the univariable and sociodemographically adjusted models, release from imprisonment was associated with increased hazards of all-cause mortality versus never-imprisonment (Model 1 adjusted HR (aHR) 1.50 (95% CI 1.17 to 1.92)). This association disappeared after further adjustment for co-occurring mental illness, drug-related harm, alcohol-related harm and CCI score (Model 2 aHR 1.02 (95% CI 0.79 to 1.31)).

**Table 4:** Associations between imprisonment history and mortality among adults with neurodevelopmental disability aged <55 years in New South Wales, 2001–2015 (n=61 431)^*^

	Univariable		Model 1^†^		Model 2^†^	
	HR (95% CI)	P value^‡^	HR (95% CI)	P value^‡^	HR (95% CI)	P value^‡^
Imprisonment history						
Not imprisoned	1.00 (ref)	0.005	1.00 (ref)	0.001	1.00 (ref)	0.903
Post-release	1.41 (1.11 to 1.78)		1.50 (1.17 to 1.92)		1.02 (0.79 to 1.31)	

* Excludes six persons with all follow-up time aged <55 years spent in prison.

† Model 1 adjusted for age, sex, Aboriginal and/or Torres Strait Islander identity, residential Index of Relative Disadvantage, residential remoteness. Model 2 further adjusted for co-occurring mental illness, drug-related harm, alcohol-related harm, Charlson Comorbidity Index.

‡ P values obtained from Wald test.

### All-cause mortality rates by time period post-release in the prison release subcohort

**Figure 3:**
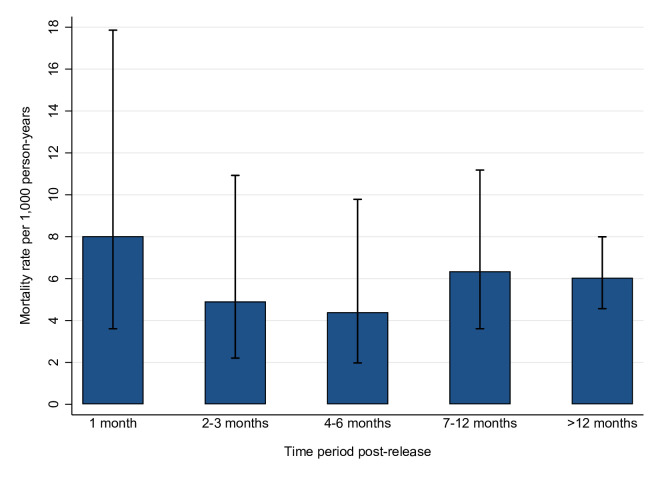
All-cause mortality rates in different time periods post-release among adults with neurodevelopmental disability released from prison in New South Wales, 2001–2015 (n=2214).

Figure 3 depicts all-cause mortality rates per 1000 person-years in different post-release time periods. All-cause mortality rate was highest in the first month post-release. However, given that the 95% CIs for all mortality rate estimates overlapped, differences in the mortality rates for each time period cannot be conclusively inferred.

### Receipt of disability support services and post-release mortality in the prison release subcohort

In Poisson regression models, being known to the prison disability service before release was associated with a higher rate of all-cause mortality post-release (unadjusted MRR 2.38 (95% CI 1.41 to 4.00), p=0.001; MRR adjusted for age, sex and Aboriginal and/or Torres Strait Islander identity 1.97 (95% CI 1.18 to 3.30), p=0.010). We found no association between receiving state-funded disability services in the same financial year as prison release and post-release all-cause mortality (unadjusted MRR 0.97 (95% CI 0.50 to 1.92), p=0.941; MRR adjusted for age, sex and Aboriginal and/or Torres Strait Islander identity 1.09 (95% CI 0.55 to 2.15), p=0.811).

## Discussion

This data-linkage cohort study among adults with neurodevelopmental disabilities in NSW, Australia, identified several key findings. First, in all age groups under 55 years, all-cause mortality rates were higher in those released from prison than those not imprisoned. External causes of death also accounted for a much greater proportion of deaths among those with a history of imprisonment. Second, after adjustment for sociodemographic factors, release from prison was associated with an increased hazard of all-cause mortality compared with never-imprisonment. However, this association disappeared after further adjusting for time-updated health characteristics, including co-occurring mental illness, drug-related and alcohol-related harm, and past-year CCI score, suggesting that these factors largely accounted for the association. Third, in the prison release subcohort, receipt of disability services in prison was associated with an increased hazard of post-release all-cause mortality. We found no association between receipt of post-release community disability services and mortality.

Strengths of our study include the use of multiple data sources to ascertain neurodevelopmental disabilities, improving sensitivity over single-source ascertainment; the large, population-based sample; and inclusion of potential sociodemographic and health-related confounders. A key limitation is representativeness and susceptibility to selection bias. This is an inherent limitation of the source datasets, which record service provision and are not a comprehensive registry of individuals with neurodevelopmental disabilities. It is likely we did not identify all NSW adults with neurodevelopmental disabilities during the study period due to underidentification of neurodevelopmental disabilities, variable service eligibility criteria, false-negative linkage errors and restricted dataset timeframes. Individuals with more significant functional impairment, who are more likely to have their disability recognised by services and/or meet service eligibility criteria, are likely to be overrepresented, and our results may not be generalisable to people with lesser functional impairment and/or barriers to service engagement. The use of educational, health and prison datasets to select our cohort may have also led to oversampling of younger individuals, individuals with health conditions and individuals with experience of imprisonment. Without knowing the true distribution of these factors among NSW adults with neurodevelopmental disability, the magnitude and direction of consequent selection bias are difficult to estimate. Our study may also be affected by some misclassification of exposures, covariates and outcomes due to linkage/clerical errors or events occurring interstate/overseas; however, we believe this is likely to be non-differential.

Our finding of elevated mortality rates among younger people with neurodevelopmental disabilities released from prison compared with their peers, and the higher contribution of external causes of death—mainly unintentional drug-induced and suicide deaths—emphasises the relevance of imprisonment as a risk factor for adverse outcomes in this group. This is not unique to people with neurodevelopmental disabilities—it is well-established in general population studies,^25^ and the previous Australian study reporting similar post-release mortality among people with and without intellectual disability^42^ suggests that the post-release period poses a high risk regardless of disability. What may differ are the support and accessibility needs of people with neurodevelopmental disabilities, and the policy and service responses needed to prevent deaths. Echoing previous research in general population^22^ and neurodevelopmental disability cohorts,^28 29^ members of our cohort who experienced imprisonment had strikingly high prevalences of co-occurring mental illness and substance-related harm. While these factors, along with physical comorbidity, largely explained associations between prison release and mortality in our study, the time-updated nature of these variables means it is unclear whether they are acting as confounders, mediators, or both. Regardless, our results highlight the importance of comprehensive physical, mental health and drug and alcohol care for people with neurodevelopmental disabilities, both in prison and post-release contexts and the broader community.

Our finding that being known to the prison disability service was associated with higher post-release mortality probably reflects the fact that people with more severe impairments are more likely to have their disability identified in prison and are at higher risk of premature death. Prison disability services should be aware of the elevated mortality risk faced by their clients post-release and ensure effective transitional planning and support. Although we did not find any association between receipt of state-funded community disability services at release and post-release mortality, we did not have information about service type or intensity, which may be important. The small size of the prison release subcohort may also have limited our statistical power. Notably, less than a quarter of this subcohort received community disability services at any prison release, suggesting scope to expand access. Given the small size of this subcohort (2214 persons over 15 years), expanded access to disability services is likely to be feasible and confer cost savings,^53^ and may produce health benefits.

Overall, our results suggest substantial unmet health and safety needs among people with neurodevelopmental disabilities released from prison. Planning and delivery of prison and post-release health and disability services should be commensurate with these needs. While NSW has made some improvements in this area, limitations remain. Specialist units established in 2006 provide education, training and pre-release planning for imprisoned men with cognitive impairment, but are limited to 57 (male only) participants at a time.^54^ Various transitional support programmes also exist, including those targeting specific needs (eg, substance use disorders), some also providing accommodation.^55^ However, they have limited capacity (with places typically prioritised based on ‘reoffending risk’) and may not be appropriate for people with disabilities regarding accessibility, intensity and/or duration.^55 56^ Previously, the NSW government funded a transitional support programme for people with intellectual disability, ranging from drop-in casework services to supported accommodation.^57^ However, there are concerns that these services have diminished after being transitioned to the NDIS (Australia’s personalised, market-based disability funding scheme introduced from 2013 to 2020).^58^ Future research should examine impacts of different disability service and transitional support approaches on post-release outcomes for this population.

Our findings also raise broader questions about how to best support people with neurodevelopmental disabilities and complex needs, including mental illness and substance use, and prevent untimely deaths. Elements proposed as important include a holistic and integrated approach,^40 59^ flexibility,^60^ strong staff-consumer relationships^61^ and effective intrasectoral and intersectoral collaboration in policy and service delivery.^40 59^ Access barriers, including system complexity,^62^ stigma^63^ and inadequate accommodation of disability by mainstream services, should also be addressed. Finally, diversion programmes may minimise imprisonment of people with neurodevelopmental disabilities and facilitate linkage to health and disability services.^64^ However, these rely on robust community-based services and must avoid placing unfair and excessive legal obligations on people with disabilities.^65^

## Conclusions

In this cohort study of adults with neurodevelopmental disabilities in NSW, we found that associations between release from imprisonment and all-cause mortality were largely explained by health-related factors, including co-occurring mental and physical health conditions and substance-related harm. We found no evidence that receipt of disability services in prison or the community were associated with reduced post-release mortality. Our findings highlight a need for holistic and integrated policy and service responses to people with neurodevelopmental disabilities who have complex needs, including criminal legal system involvement, mental illness and substance use.

## Supplementary material

10.1136/bmjopen-2025-102805 online supplemental file 1

## Data Availability

No data are available.
