# Supplementary material for: Imprisonment and mortality among adults with neurodevelopmental disabilities in New South Wales, Australia, 2001–2015: a data-linkage cohort study
Source: BMJ Open. 2025 Oct 13;15(10):e102805. doi: 10.1136/bmjopen-2025-102805 (PMC12519720; doi:10.1136/bmjopen-2025-102805)
Supplement: online supplemental file 1 [file bmjopen-15-10-s001.docx]

**Supplemental material**

**Table of Contents**

[**Criteria for identifying cases of false-positive linkage error** 2](#_Toc205477247)

[**Table S1. Full model results for Cox regressions examining associations between imprisonment history and mortality among adults with neurodevelopmental disability aged <55 years in NSW, 2001-2015 (n=61,431)** 3](#_Toc205477248)

## **Criteria for identifying cases of false-positive linkage error**

Criteria for identifying cases of possible linkage error are outlined below. If any participant met any of the below criteria, it was considered likely that the participant had one or more false positive linkage errors and/or that key data was unreliable, and the participant was excluded from the study.

| **Dataset** | **Criteria for identifying linkage error** |
| --- | --- |
| Admitted Patient Data Collection (APDC) | - Birth date >44 weeks before start date (to account for any records created for unborn children) - Age >116 years at episode start date or episode end date - Date of death >3 days before episode start date - Date of death >28 days before episode end date |
| Emergency Department Data Collection (EDDC) | - Birth date >44 weeks before arrival date (to account for any records created for unborn children) - Age >116 years at arrival date or departure date - Mode of separation is not “dead on arrival” and death date is >3 days before arrival date - Mode of separation is not “dead on arrival” and death date is >6 days before departure date - Mode of separation is “dead on arrival” and death date is >365 days before arrival date - Mode of separation is “dead on arrival” and death date is >368 days before departure date |
| Mental Health Ambulatory Data Collection (MH-AMB) | - Birth date after service contact date - Age >116 years at service contact date - Death date >14 days before service contact date |
| Offender Integrated Management System (OIMS) | - Age <7 years at episode start date or episode end date - Age >116 years at episode start date or episode end date - Death date < episode start date - Death date >3 days before episode end date - Episode start or end dates overlap by >1 day |
| Disability Services Minimum Dataset (DS-MDS) | - Birth date after end of financial year in which services were received - Age >116 years at start of financial year in which services were received - Age >116 years at end of financial year in which services were received - Death date >28 days before start of financial year in which services were received |
| Statewide Disability Services (SDS) dataset | - Age >116 at SDS referral date - Death date >3 days before SDS referral date - Age <7 years at SDS referral date |
| Additional demographic criteria | - Age >116 years at death - Age >116 years at 30 June 2015 |

## **Table S1. Full model results for Cox regressions examining associations between imprisonment history and mortality among adults with neurodevelopmental disability aged <55 years in NSW, 2001-2015 (n=61,431)^a^**

|  | Univariable | | Model 1^b^ | | Model 2^b^ | |
| --- | --- | --- | --- | --- | --- | --- |
|  | HR (95% CI) | p value ^c^ | HR (95% CI) | p value ^c^ | HR (95% CI) | p value ^c^ |
| Imprisonment history |  |  |  |  |  |  |
| *Not imprisoned* | 1.00 (ref) | 0.005 | 1.00 (ref) | 0.001 | 1.00 (ref) | 0.903 |
| *Post-release* | 1.41 (1.11-1.78) |  | 1.50 (1.17-1.92) |  | 1.02 (0.79-1.31) |  |
| Sex |  |  |  |  |  |  |
| *Male* |  |  | 1.00 (ref) | 0.006 | 1.00 (ref) | 0.002 |
| *Female* |  |  | 0.88 (0.80-0.96) |  | 0.86 (0.79-0.95) |  |
| Age group |  |  |  |  |  |  |
| *18-24 years* |  |  | 1.00 (ref) | <0.001 | 1.00 (ref) | <0.001 |
| *25-34 years* |  |  | 1.29 (1.08-1.54) |  | 1.24 (1.04-1.49) |  |
| *35-44 years* |  |  | 2.14 (1.81-2.54) |  | 1.92 (1.62-2.27) |  |
| *45-54 years* |  |  | 4.12 (3.50-4.84) |  | 3.39 (2.88-3.99) |  |
| Index of Relative Socioeconomic Disadvantage |  |  |  |  |  |  |
| *Lowest quintile* |  |  | 1.00 (ref) | <0.001 | 1.00 (ref) | 0.004 |
| *Second lowest quintile* |  |  | 1.02 (0.89-1.17) |  | 1.04 (0.91-1.19) |  |
| *Middle quintile* |  |  | 0.76 (0.66-0.87) |  | 0.82 (0.71-0.94) |  |
| *Second highest quintile* |  |  | 0.89 (0.77-1.03) |  | 0.95 (0.82-1.10) |  |
| *Highest quintile* |  |  | 0.79 (0.67-0.92) |  | 0.85 (0.73-1.00) |  |
| Remoteness area |  |  |  |  |  |  |
| *Major Cities* |  |  | 1.00 (ref) | 0.072 | 1.00 (ref) | 0.419 |
| *Inner Regional* |  |  | 0.96 (0.86-1.08) |  | 0.99 (0.89-1.11) |  |
| *Outer Regional/Remote* |  |  | 0.82 (0.69-0.97) |  | 0.89 (0.75-1.06) |  |
| Co-occurring mental illness |  |  |  |  |  |  |
| *No* |  |  |  |  | 1.00 (ref) | <0.001 |
| *Yes* |  |  |  |  | 1.26 (1.13-1.41) |  |
| Drug-related harm |  |  |  |  |  |  |
| *No* |  |  |  |  | 1.00 (ref) | 0.689 |
| *Yes* |  |  |  |  | 1.03 (0.88-1.21) |  |
| Alcohol-related harm |  |  |  |  |  |  |
| *No* |  |  |  |  | 1.00 (ref) | <0.001 |
| *Yes* |  |  |  |  | 1.46 (1.25-1.71) |  |
| Charlson Comorbidity Index score |  |  |  |  |  |  |
| *0* |  |  |  |  | 1.00 (ref) | <0.001 |
| *≥1* |  |  |  |  | 10.51 (9.38-11.78) |  |

HR=Hazard ratio. CI=Confidence interval. ^a^Excludes 6 persons with all follow up time aged <55 years spent in prison. ^b^Model 1 adjusted for age, sex, Aboriginal and/or Torres Strait Islander identity, residential Index of Relative Disadvantage, residential remoteness. Model 2 further adjusted for co-occurring mental illness, drug-related harm, alcohol-related harm, Charlson Comorbidity Index. ^b^p values obtained from Wald test.
