# Supplementary material for: Imprisonment and mortality among adults with neurodevelopmental disabilities in New South Wales, Australia, 2001–2015: a data-linkage cohort study
Source: BMJ Open. 2025 Oct 13;15(10):e102805. doi: 10.1136/bmjopen-2025-102805 (PMC12519720; doi:10.1136/bmjopen-2025-102805)
Supplement: Supplementary file 2 [file bmjopen-2025-102805.reviewer_comments.pdf]

PEER REVIEW HISTORY

BMJ Open publishes all reviews undertaken for accepted manuscripts. Reviewers are asked to complete a checklist review form and are provided with free text boxes to elaborate on their assessment. These free text comments are reproduced below.

ARTICLE DETAILS

Title (Provisional)

Imprisonment and mortality among adults with neurodevelopmental disabilities in NSW, Australia, 2001-2015: a data-linkage cohort study

Authors

Spike, Erin; Srasuebkul, Preeyaporn; Kariminia, Azar; Butler, Tony; Trollor, Julian

---

VERSION 1 - REVIEW

**Reviewer**	**1**
**Name**	**Thygesen, Lau**
**Affiliation**	**Syddansk Universitet**
**Date**	**28-Jun-2025**
**COI**	**None**

Review of paper by Spike and colleagues: "Imprisonment and mortality among adults with intellectual and other neurodevelopmental disabilities in NSW, Australia, 2001-2015: a retrospective data-linkage cohort study

Thank you for the opportunity to read this interesting paper of a vulnerable group of persons with neurodevelopmental disabilities in Australia. I think the methods section is detailed and the paper shows interesting results. I have a few major comments and several minor comments for the authors' consideration.

Major comments:

The objective of the study is clearly written in the introduction section, but when I read the abstract the first time, I did not really get the idea of only including people with ID and other neurodevelopmental disorders. I guess the main idea is to make the groups more comparable, but I would recommend that this argument is more clearly presented in the abstract or maybe further developed in the introduction.

In the introduction several references are made to First Nation people even though it is not clear why this element is highlighted in the present study (focusing on people with neurodevelopmental disorders). Furthermore, due to privacy restrictions, you are not

allowed to make descriptive results of this subpopulation in the analyses. I would recommend removing these elements in the introduction.

The presentation of the probabilistic merged datasets has many details. I wondered how well the data reflect the total population throughout the whole period 2001-2015 – it seems like some of the datasets only include data from part of the period. I think this should be highlighted either in the methods section or discussion. Another element is the validity of the probabilistic record linkage – how good is the linkage (proportion of false positives and false negatives)? I would include this as part of the discussion of limitations.

It seems like you compare persons with neurodevelopmental disorders in prison with those not in prison throughout the period 2001-2015. This to some degree means that you condition on the future – a more appropriate approach would be to include all persons from start of follow-up and then include imprisonment as a time-varying exposure.

Another element is that you remove mortality during imprisonment (which (a bit surprising) is low) thereby you actually overestimate the mortality risk in the exposed group.

Minor comments:

I am not fond of using the term retrospective since the data included was prospectively administered when it was included. I prefer something like 'historical cohort study' or cohort study using already collected data (or something similar).

Follow-up only includes information on mortality. I wondered if any data on migration (out of Australia) is available since this will underestimate mortality rates.

One month equals 28 days – why not use 30 (or 31) days since that is more like the average (except February).

Table titles lack some information, e.g. which cohort is included (only people with neurodevelopmental disorders) and which period. Table 4 includes many numbers – maybe only include the relevant results from the exposure of interest?

I appreciate the discussion, which highlights the importance of close monitoring of the cohort after release from prison. I think the discussion should include some of the potential limitations listed above in this review.

Reviewer 2

Name Abuga, Jonathan

Affiliation Kenya Medical Research Institute Wellcome Trust Research Programme, Clinical Research (Neurosciences)

Date 06-Jul-2025

COI **None**

An important paper about mortality outcomes comparing imprisoned individuals with neurodevelopmental disabilities compared to those not imprisoned with the same disabilities, in NSW, Australia. The paper on an under-investigated yet important topic would benefit should the authors consider the following comments and suggestions:

1. In the Abstract, the Methods and Materials are conspicuously missing. In the results, what is the overall/crude estimate of mortality in all age groups? Appropriate measures (s) of mortality, like rate/risk ratio, can be reported.

2. I have provided additional references, including a systematic review, to have a more diverse and inclusive literature overview on neurodevelopmental disabilities and mortality outcomes for the introduction section, lines 74-77 (https://pubmed.ncbi.nlm.nih.gov/33897590/; https://pubmed.ncbi.nlm.nih.gov/31653591/;)

Data sources and linkage

3. It would be worth mentioning whether there was a unique identifier, such as a "patient number," for the participants using the listed services from the various data sources, thereby providing a more justifiable reason for performing the probabilistic data linkage. Please clarify.

4. Please note, the various data sources have different start and end dates, or duration for which data was available or collected; I wonder why the authors have not clarified the specific dates covered by their retrospective data linkage cohort study at the earliest opportunity. Please revise

5. There is a need for clarity on the definition of "neurodevelopmental disability" in the current study. Whether it had to be a primary diagnosis or a secondary diagnosis based on the records has to be clarified as well.
6. How was imprisonment determined? Did imprisonment come before or after the diagnosis of neurodevelopmental disability? Please clarify within the text.

7. Please provide some further details about data linkage, for example, the specific criteria or algorithm used, and please clarify the suspected linkage errors.

The Main exposure

8. There is some sort of disconnection between imprisonment and neurodevelopmental disabilities diagnosis in the way the information is presented in the text. Here, the authors do not explain how classification of participants was done based on the main exposure (that is, those with disability and imprisoned—the cases, versus controls—those with disability but not imprisoned). How were cases and controls (the comparison groups) selected from the data linkage study? Please clarify.

9. For the Cox proportional Hazards regression model, please clarify 3 issues: (i) the baseline of the time-to-event (all-cause mortality) modeling; (ii) Censoring, if any was done, and (iii) if all the basic assumptions for the models were met—please mention these assumptions and how they were checked during data analysis.

10. Also, provide the rationale for the two separate adjusted models, and why not a single adjusted model. Please clarify.

11. In Line 272, there were 9,995 releases in total, for 2,300 individuals who were imprisoned. Does this mean multiple imprisonments for the same individuals to warrant multiple releases? This information should be provided in the Methods section as well. Refer to my comments #6 and 8 above.

12. Lines 273-285, how different are those proportions statistically? The authors need to provide p-values for the difference in proportions/percentages.

13. Table 2: Regarding “NR”, I wonder why it was not possible to state the numbers (n), even if they were less than 5. I do not understand what “back-calculation of cells” means. I am not convinced that it is impossible to get these values, given that the total (N) in each category is known. Please clarify.

14. What does the overlapping of confidence intervals mean? (Lines 332-333). Based on this information, all–cause mortality was not different (or were similar) for the various months post-release in Figure 3. Please revise this paragraph.

15. Strengths and limitations of the study should come after discussing the key findings, just before the conclusion.

16. Regarding selection bias, please clarify if selection bias originates from the nature of all the datasets available for use, or from the identification and selection process of the datasets used in the current study.

17. The modelling of time-varying covariates should be expressly mentioned in the methods first, before appearing in the discussion. Refer to my comment #10 above.

18. To make the title more concise, the phrase "neurodevelopmental disabilities" would suffice for "intellectual and other neurodevelopmental disabilities", even if most study participants had intellectual disability.

VERSION 1 - AUTHOR RESPONSE

Reviewer: 1
Dr. Lau Thygesen, Syddansk Universitet, Nationale Institute of Public Health, University of Southern Denmark

Comments to the Author:
Review of paper by Spike and colleagues: "Imprisonment and mortality among adults with intellectual and other neurodevelopmental disabilities in NSW, Australia, 2001-2015: a retrospective data-linkage cohort study

Thank you for the opportunity to read this interesting paper of a vulnerable group of persons with neurodevelopmental disabilities in Australia. I think the methods section is detailed and the paper shows interesting results. I have a few major comments and several minor comments for the authors' consideration.

Major comments:

The objective of the study is clearly written in the introduction section, but when I read the abstract the first time, I did not really get the idea of only including people with ID and other neurodevelopmental disorders. I guess the main idea is to make the groups more comparable, but I would recommend that this argument is more clearly presented in the abstract or maybe further developed in the introduction.

Thank you for the suggestion to clarify the rationale for the study. The reason for conducting this study within a cohort of people with neurodevelopmental disability is that people with neurodevelopmental disabilities are a population with distinct experiences of marginalisation and discrimination, specific and often complex health and support needs, and distinct interactions with service systems (e.g., use of both health and disability services). Imprisonment may or may not have a differential association with mortality within this population compared to the general population. This relates to comparability, but also to the fact that people with neurodevelopmental disabilities are a distinct population in terms of policy, service and program design. It is therefore important to consider risk factors for adverse outcomes within this group specifically.

We have revised the 'Objectives' section to make the rationale clearer as follows (abstract lines 17 to 20):

Previous text:

Objectives: 1) In adults with neurodevelopmental disabilities, compare mortality between those who have and have not experienced imprisonment; 2) In adults with neurodevelopmental disabilities who have been released from prison, examine associations between receipt of disability services and post-release mortality.

Updated text:

Objectives: *1) Examine associations between imprisonment history and mortality among adults with neurodevelopmental disabilities; 2) Examine associations between receipt of disability services and post-release mortality among adults with neurodevelopmental disabilities released from prison.*

We have also added the following sentences to the introduction (lines 80 to 84) to make the rationale clearer:

However, there is little evidence regarding the extent to which imprisonment is associated with health outcomes – including mortality – among people with neurodevelopmental disability. This is important given the distinct needs, service interactions, and patterns of marginalisation experienced by this population.

In the introduction several references are made to First Nation people even though it is not clear why this element is highlighted in the present study (focusing on people with neurodevelopmental disorders). Furthermore, due to privacy restrictions, you are not allowed to make descriptive results of this subpopulation in the analyses. I would recommend removing these elements in the introduction.

We have removed this information from the first paragraph of the introduction as suggested by the reviewer. We have, however, retained and revised the following text in the introduction (lines 88 to 91):

In Australia, inadequate disability recognition, supports and accommodations have been noted throughout criminal legal systems, including prisons, with a lack of culturally appropriate disability services for First Nations people, who are incarcerated at 15 times the rate of other NSW adults.

While not the focus of this paper, we believe that the extreme overrepresentation of First Nations people in NSW prisons and the lack of culturally appropriate services are important aspects of the context in which this study took place.

The presentation of the probabilistic merged datasets has many details. I wondered how well the data reflect the total population throughout the whole period 2001-2015 – it seems like some of the datasets only include data from part of the period. I think this should be highlighted either in the methods section or discussion.

The variable timeframes covered by each dataset are an inherent limitation of the data collections we used – as they are independent datasets collected by different organisations, they commenced at different times. They also have different lag times in terms of the most recent data that can be extracted.

All datasets had data available until the end of the study period (30 June 2015) except the Australian Co-ordinating Registry's Cause of Death Unit Record File (COD-URF) containing both cause and fact of death information. However, fact of death was also recorded in the NSW Registry of Births, Deaths and Marriages Death Registrations dataset, for which we did have data available through to 30 June 2015.

To highlight this issue more clearly, we have added the following sentence to the methods section (lines 112 to 113):

Dataset commencement and end dates differed, but most had complete coverage for the study period of 1 July 2001 – 31 June 2015.

The specific datasets which did not have complete coverage are listed below. We address and justify each in turn, although it is not possible to do this in the manuscript due to space constraints:

- the NSW Department of Ageing, Disability and Home Care Disability Services Minimum Dataset (DS-MDS), recording state-funded disability service provision [July 2005 – June 2016] – This data was used for both cohort selection and for ascertainment of the post-release disability service use variable (one of the exposures of interest). We acknowledge that using this variable prior to July 2005 would introduce bias into our study, as no individuals would have post-release disability service use recorded during this period. We have therefore adjusted the presentation/analysis of our data, as follows:
    - In Table 1, for the proportion of persons who ever received community disability services during follow up, we have added a footnote to explicitly state that the denominator includes 941 persons who died on/before 01 July 2005 (see Table 1 footnote c, lines 275 to 276). We have not excluded these persons because there were n<5 persons lost from the 'imprisoned during follow up' column, and excluding these persons would mean that the exact number could be back-calculated. Our ethics approvals do not permit presentation of data with numbers <5 or where this can be back-calculated. Excluding these persons results in changes to the presented percentages of <1 percentage point.
    - In Table 2, however, we have included a similar footnote for the corresponding variable (see Table 2 footnote c, lines 297-298).
    - Also in Table 2, we restricted analysis of the proportion of persons who received community disability services at any prison release to persons with one or more prison releases occurring on/after 01 July 2005 (see Table 2 footnote d, lines 297 to 299).
    - In the Poisson regression models analysing adjusted and unadjusted associations between receipt of community disability services at any prison release and post-release mortality, we also restricted the analytic sample to

persons with one or more prison releases occurring on/after 01 July 2005, with no substantial changes to the results (see methods line 250 to 252).

- the NSW Ombudsman dataset, recording reviewable residential care deaths [December 2002 – December 2015] – This data was used for cohort selection only. The incomplete coverage of this dataset for 18 months at the commencement of our study period could mean that some otherwise eligible individuals – all of whom would have died during our study period – were not identified. However, this is likely to be a small number which is unlikely to substantially alter our results.
- the NSW Disability Programs in Public Schools dataset, recording disability service provision within public schools [January 2011 – June 2015] – This dataset was used for cohort selection only. The incomplete coverage of this dataset may have led to disproportionate inclusion of younger individuals towards the end of our study period (i.e., individuals who were school-aged between January 2011 – June 2015 who subsequently turned 18 and thereby became eligible for inclusion in our cohort). However, we adjust for age as a covariate and also believe that this is unlikely to substantially alter our results.
- the Emergency Department Data Collection (EDDC), recording emergency department presentations to public hospitals [Jan 2005 – June 2016] – This dataset was used for both cohort selection and ascertainment of two covariates (drug-related harm and alcohol-related harm). In terms of cohort selection, we believe it is unlikely that many individuals would have their neurodevelopmental disability identified solely in the emergency department, i.e., they are likely to also have their disability identified in another health or disability service dataset (and therefore would still appear in our cohort). In terms of covariates, the EDDC was one of three sources (the others being the APDC and MH-AMB) used to ascertain drug-related harm and alcohol-related harm. As the other two data sources had data available for the entire period, we believe the degree of under-ascertainment for these variables for the first 3.5 years of the study is likely to be minimal.
- the Australian Co-ordinating Registry's Cause of Death Unit Record File (COD-URF) recording deaths and causes of deaths in NSW [January 1985 December 2013] – as described above, this was one of two sources used to obtain fact of death information, and the other source (RBDM-DR) had complete coverage for the study period. We also report cause of death information, but we clearly state that this is restricted to deaths for which this information is available (Table 3, lines 315 to 320).

The reviewer specifically mentioned representativeness as a concern. Ideally, we would have complete coverage for all cohort-ascertainment datasets for a prolonged period (i.e., covering the lifespan of all individuals in the study), however our sample would still be more reflective of those individuals with greater functional impairment who are more likely to have their disability known to services. We have edited the strength and limitations section of our discussion to address the issue of representativeness more clearly as follows (lines 366 to 379):

A key limitation is representativeness and susceptibility to selection bias. This is an inherent limitation of the source datasets, which record service provision and are not a comprehensive registry of individuals with neurodevelopmental disabilities. It is likely we did not identify all NSW adults with neurodevelopmental disabilities during the study period due to under-identification of neurodevelopmental disabilities, variable service eligibility criteria, false-negative linkage errors, and restricted dataset timeframes. Individuals with more significant functional impairment, who are more likely to have their disability recognised by services and/or meet service eligibility criteria, are likely to be overrepresented, and our results may not be generalisable to people with lesser functional impairment and/or barriers to service

engagement. The use of educational, health and prison datasets to select our cohort may have also led to oversampling of younger individuals, individuals with health conditions, and individuals with experience of imprisonment. Without knowing the true distribution of these factors among NSW adults with neurodevelopmental disability, the magnitude and direction of consequent selection bias is difficult to estimate.

Another element is the validity of the probabilistic record linkage – how good is the linkage (proportion of false positives and false negatives)? I would include this as part of the discussion of limitations.

We have added further detail to the methods section describing the validity of the linkage method and estimated proportion of false positives and negatives as follows, including an updated reference with additional detail (lines 136 to 138):

The linkage method uses probability weighting supplemented by manual review of intermediate-probability matches, with estimated false-positive and false-negative rates of 0.5%, and the linkage procedure undergoes regular quality assurance checks.[1]

The number of likely false-positive linkage results identified in our study, which we identified using our own post-linkage quality assurance checks (i.e. comparing event dates recorded in datasets to birth/death dates) is consistent with the 0.5% target (341/67,217 = 0.51%). It is not possible for us to check for false-negative results as these records would not have been identified by the linkage centre, and therefore would not be included in the de-identified data we received.

We do not believe that the small proportions of false positive and negative linkage results are likely to substantially affect our results.

We have added specific mention of linkage errors in our discussion as a factor which may introduce selection bias and affect the representativeness of our sample (line 371). We had also already mentioned that linkage errors could lead to misclassification of exposures, covariates or outcomes, but that we believe this is likely to be non-differential (lines 380 to 382).

It seems like you compare persons with neurodevelopmental disorders in prison with those not in prison throughout the period 2001-2015. This to some degree means that you condition on the future – a more appropriate approach would be to include all persons from start of follow-up and then include imprisonment as a time-varying exposure.

We did use the latter approach (i.e., imprisonment history was already included as a time-varying variable in our analysis) and had already noted that imprisonment history was a time-updated variable. However, we acknowledge that this could have been more clearly explained. We have revised the wording of the methods section to make this clearer (line 179 to 182 with the most relevant part bolded):

The main exposure was imprisonment history, i.e. any imprisonment (remand or sentenced) and subsequent release from a NSW adult prison during the study period, ascertained from imprisonment start and end dates recorded in OIMS. ***This was treated as a time-updated variable (never imprisoned/in prison/post-release).***

Another element is that you remove mortality during imprisonment (which (a bit surprising) is low) thereby you actually overestimate the mortality risk in the exposed group.

It is correct that we would overestimate the total effect of imprisonment and release on mortality. However, we believe it makes more sense to treat imprisonment and release from imprisonment as separate levels of exposure due to the extreme differences between prison and community environments, which are reflected in extreme differences in mortality risk. For example, the US study by Spaulding and colleagues we cited to justify this claim reported an SMR of 0.85 for people in prison (compared to the general population), versus an SMR of 1.54 for people released from prison.[2] Similarly, in a previous Australian study, the SMR among imprisoned men (compared to men in the general community) was 1.7 in prison versus 4.0 post-release, and for women was 2.1 in prison versus 8.2 post-release, indicating much higher mortality in prison versus post release.[3]

While this might seem counter-intuitive, imprisonment is a highly artificial, controlled and surveilled environment in which many common hazards are restricted. For example, the risk of transport accidents is almost eliminated. Access to illicit drugs (while not eliminated) is also likely to be partially restricted, and access to some means of suicide (e.g. firearms, large quantities of medication) is also heavily restricted. Although suicide, overdose and violent deaths occur in prison, many prisons take considerable steps to prevent these, including intensive surveillance. In saying this, we do not wish to minimise in any way the moral, ethical and legal concerns raised by deaths in custody and the adverse impacts of these deaths on individuals, families and communities.

While we ideally would have included an analysis of the association between current imprisonment and mortality in our cohort, the small number of deaths (n<10) made this unfeasible, and we believe excluding the time in prison from follow up as we have done is a reasonable alternative.

Minor comments:

I am not fond of using the term retrospective since the data included was prospectively administered when it was included. I prefer something like ‘historical cohort study’ or cohort study using already collected data (or something similar).

We acknowledge that the term ‘retrospective’ can be confusing, as outlined in the STROBE guideline explanation and elaboration paper.[4] We have reworded the ‘design’ subheading of the abstract (line 21) to state:

Population-based data-linkage cohort study using historical administrative data.

We have likewise amended the first line of our methods section (lines 111 to 112) to state:

We performed a data-linkage cohort study using historical data from multiple NSW administrative datasets.

We have also removed use of the word ‘retrospective’ elsewhere in our manuscript, including the title.

Follow-up only includes information on mortality. I wondered if any data on migration (out of Australia) is available since this will underestimate mortality rates.

Unfortunately we do not have any data on migration out of Australia.

One month equals 28 days – why not use 30 (or 31) days since that is more like the average (except February).

We have amended our analysis to make one month equivalent to 30 days (see methods line 246). This has not resulted in any substantial changes to Figure 3.

Table titles lack some information, e.g. which cohort is included (only people with neurodevelopmental disorders) and which period.

We have amended the table titles as follows:

Table 1. Descriptive characteristics of adults with neurodevelopmental disabilities in NSW 2001–2015 by imprisonment status during follow up

Table 2. Descriptive characteristics of adults with neurodevelopmental disabilities in NSW released from prison from 2001–2015 by survival status at end of follow up

Table 3. Cause of death by imprisonment history during follow up among adults with neurodevelopmental disability aged <55 years at death in NSW, 2001-2015

Table 4. Associations between imprisonment history and mortality among adults with neurodevelopmental disability aged <55 years in NSW, 2001-2015 (n=61,431)

Table 4 includes many numbers – maybe only include the relevant results from the exposure of interest?

We agree, and have placed the full results table in Supplemental material (see Table 4 lines 329 to 334 and Supplemental material, Supplementary Table S1).

I appreciate the discussion, which highlights the importance of close monitoring of the cohort after release from prison. I think the discussion should include some of the potential limitations listed above in this review.

We have amended the limitations section of our discussion as outlined above.

Reviewer: 2
Dr. Jonathan Abuga, Kenya Medical Research Institute Wellcome Trust Research Programme, Kisii University

Comments to the Author:
An important paper about mortality outcomes comparing imprisoned individuals with neurodevelopmental disabilities compared to those not imprisoned with the same disabilities, in NSW, Australia. The paper on an under-investigated yet important topic would benefit should the authors consider the following comments and suggestions:

1. In the Abstract, the Methods and Materials are conspicuously missing. In the results, what is the overall/crude estimate of mortality in all age groups? Appropriate measures (s) of mortality, like rate/risk ratio, can be reported.

We have added a methods section to our abstract (lines 25 to 28). We did not include one originally as it is not one of the required abstract subheadings according to the BMJ Open author guidelines, however we agree that this improves our abstract.

We have amended Figure 2 to include the overall mortality rate for each age group. We have not reported rate/risk ratios as we have used Cox regression to compare the mortality outcomes of those exposed and not exposed to imprisonment.

2. I have provided additional references, including a systematic review, to have a more diverse and inclusive literature overview on neurodevelopmental disabilities and mortality outcomes for the introduction section, lines 74-77 (https://pubmed.ncbi.nlm.nih.gov/33897590/; https://pubmed.ncbi.nlm.nih.gov/31653591/;)

We thank the reviewer for these suggestions and have included the systematic review as an additional citation.

Data sources and linkage

3. It would be worth mentioning whether there was a unique identifier, such as a "patient number," for the participants using the listed services from the various data sources, thereby providing a more justifiable reason for performing the probabilistic data linkage. Please clarify.

Unfortunately there is no unique patient identifier that can be used to link participants across the different datasets, which is why deterministic linkage was not possible. For brevity, we think it is reasonable not to state this explicitly in the manuscript, as we believe most readers would assume that probabilistic linkage is undertaken in situations where deterministic linkage is not possible. We have provided an updated reference with more details of the linkage methods used by the data linkage centre (the NSW Health Centre for Health Record Linkage) for interested readers.

4. Please note, the various data sources have different start and end dates, or duration for which data was available or collected; I wonder why the authors have not clarified the specific dates covered by their retrospective data linkage cohort study at the earliest opportunity. Please revise.

A similar point was raised by the first peer reviewer. To highlight this issue more clearly, we have added the following sentence to the methods (lines 112 to 113):

Dataset commencement and end dates differed, but most had complete coverage for the study period of 1 July 2001 – 31 June 2015.

In our above response to the first peer reviewer, we have specifically addressed each dataset which did not have complete coverage for the study period and justified why we think the potential for bias remains acceptably minor.

5. There is a need for clarity on the definition of "neurodevelopmental disability" in the current study. Whether it had to be a primary diagnosis or a secondary diagnosis based on the records has to be clarified as well.

We have added the following sentence to the start of our introduction to provide a conceptual definition of neurodevelopmental disability (lines 63 to 66):

Neurodevelopmental disabilities refer to differences in brain processing and consequent functional impairments associated with a range of childhood-onset conditions. These include intellectual disability, autism, attention-deficit hyperactivity disorder (ADHD), and fetal alcohol spectrum disorders (FASD).

We have also provided considerable detail about how neurodevelopmental disabilities were ascertained, including the use of both primary and secondary diagnoses, in the methods section (lines 154 to 164). We believe this information is sufficiently detailed given the space constraints inherent to a journal article. We have also cited references to our previously published studies which provide additional detail (line 154).

6. How was imprisonment determined? Did imprisonment come before or after the diagnosis of neurodevelopmental disability? Please clarify within the text.

As noted in the methods section (lines 162 to 164), because neurodevelopmental disabilities have a childhood onset by definition (though they may remain undiagnosed until adulthood) and typically involve a degree of persistent impairment, we assume that neurodevelopmental disability commenced prior to the 18th birthday even if it is first recorded in one of the source datasets after this age. This is consistent with the approach taken in our previous data-linkage research.[5–7] Therefore, imprisonment could occur at any time during the study period, irrespective of whether this is before or after the first record of neurodevelopmental disability in one of the source datasets. We have only included adult imprisonments (i.e. imprisonments after the onset of neurodevelopmental disability, assuming a childhood onset; as stated in the methods section line 184 to 185).

We have added further detail to our description of how imprisonment history was ascertained, which was also partly in response to comments from Reviewer 1, as follows (lines 179 to 185):

The main exposure was imprisonment history, i.e. any imprisonment (remand or sentenced) and subsequent release from a NSW adult prison during the study period, ascertained from imprisonment start and end dates recorded in OIMS. This was treated as a time-updated variable (never imprisoned/in prison/post-release). As mortality is lower in prison versus community settings, and due to few in-prison deaths in our cohort (n<10), time in prison was excluded from follow up. As our interest was in adult imprisonments, we excluded imprisonments wherein a person was aged <18 years for the entirety.

7. Please provide some further details about data linkage, for example, the specific criteria or algorithm used, and please clarify the suspected linkage errors.
We have added further detail about the linkage method, and an updated reference with more details of the linkage methods used by the data linkage centre (the NSW Health Centre for Health Record Linkage) (lines 134 to 138):

The NSW Health Centre for Health Record Linkage (CHeReL) performed probabilistic linkage between datasets with Choicemaker software, using names, birthdates, and addresses. The linkage method uses probability weighting supplemented by manual review of intermediate-probability matches, with estimated false-positive and false-negative rates of 0.5%, and undergoes regular quality assurance checks.[1]

We have also created a Supplemental material file which provides further details of the criteria used to identify suspected linkage error (referred to in methods line 168).

The Main exposure

8. There is some sort of disconnection between imprisonment and neurodevelopmental disabilities diagnosis in the way the information is presented in the text. Here, the authors do not explain how classification of participants was done based on the main exposure (that is, those with disability and imprisoned—the cases, versus controls—those with disability but not imprisoned). How were cases and controls (the comparison groups) selected from the data linkage study? Please clarify.

We thank the reviewer for prompting us to clarify this point. As noted above, selection into the cohort was based on 1) the presence of neurodevelopmental disability and 2) age- and time-period criteria (i.e., being alive and >=18 years for any part of the study period of 1 July 2001 to 31 June 2015). Selection into the cohort was not based on imprisonment status,

which was a time-updated exposure variable. We have stated the cohort eligibility criteria in lines 150 to 153 of the methods section. For greater clarity, we have also moved the information on when participants entered and exited observation to directly after the mention of eligibility criteria (153 to 154). The relevant section now reads:

Our study population comprised all adults with intellectual disability or autism recorded in any disability or health dataset, or any other neurodevelopmental disability recorded in the APDC or MH-AMB, who were alive and aged ≥18 years at any time between 1 July 2001 and 30 June 2015. Participants entered observation on the later of 1 July 2001 or their 18th birthday, with follow up until 30 June 2015 or death.

9. For the Cox proportional Hazards regression model, please clarify 3 issues: (i) the baseline of the time-to-event (all-cause mortality) modeling; (ii) Censoring, if any was done, and (iii) if all the basic assumptions for the models were met—please mention these assumptions and how they were checked during data analysis.

The baseline hazard function is the hazard function for the unexposed group (i.e. those with no imprisonment history). This is made clear in Table 4 by stating that the non-imprisoned group is the reference category for the main exposure (lines 329-334). For all participants, follow up was censored at the earlier of death or 30 June 2015 (as stated in the methods line 153 to 154); there was no left-censoring. Follow up time during imprisonment was excluded (as stated in the methods lines 183 to 184). We have added a sentence to the methods section to describe how we verified the proportional hazards assumption (lines 241 to 243):

We verified the proportional hazards assumption for each model graphically using Kaplan-Meier, log-log and Schoenfeld residual plots.

10. Also, provide the rationale for the two separate adjusted models, and why not a single adjusted model.

We believe it is reasonable to perform progressive adjustment for a) sociodemographic factors and b) health-related factors as two thematically different sets of potential confounders. Rather than presenting one maximally adjusted model, this approach allows the reader to appreciate the additional confounding effect of health-related factors in conjunction with sociodemographic factors. It also increases comparability of our study to other research, given that it is common practice to adjust for sociodemographic confounders, whereas health-related confounders are variably adjusted for. We do not provide a detailed justification for this in the manuscript due to space constraints.

11. In Line 272, there were 9,995 releases in total, for 2,300 individuals who were imprisoned. Does this mean multiple imprisonments for the same individuals to warrant multiple releases? This information should be provided in the Methods section as well. Refer to my comments #6 and 8 above.

Yes, multiple imprisonments/releases could be included for the same individual. This does not affect the Cox regression models (as once an individual is categorised as 'post-release', they remain in this category until the end of follow up, excluding any subsequent time in prison, as stated in the methods section line 179 to 185). However, we have stated this explicitly in relation to the prison and post-release disability service variables (lines 219 to 225 with relevant part bolded):

In the prison-release sub-cohort, we measured whether a person was known to the prison disability service at the time of release (a proxy for receipt of disability services in prison), based on whether they had previously appeared as a client in the SDS dataset. We also

measured receipt of state-funded community disability services at the time of release using the DS-MDS. As the DS-MDS aggregated this information by financial year, disability services were assumed to be present at release if they were recorded in the same financial year. ***If multiple releases occurred, these variables were time-updated at each release****.*

We have also stated this explicitly in relation to the Poisson regression models (lines 246 to 250 with relevant part bolded):

To examine associations between receipt of disability services (during imprisonment and post-release) and post-release all-cause mortality, we modelled mortality rate ratios (MRRs) using random-effects Poisson regression (taking post-release person-years as the exposure time and adjusting standard errors for within-participant clustering ***to account for multiple releases****).*

12. Lines 273-285, how different are those proportions statistically? The authors need to provide p-values for the difference in proportions/percentages.
This is presented as descriptive information only to highlight differences in the characteristics of the exposed and unexposed groups. We have not examined these differences statistically as our main exposure of interest is imprisonment history, and believe it is reasonable to avoid performing excessive hypothesis testing which increases the chance of type I error.

13. Table 2: Regarding "NR", I wonder why it was not possible to state the numbers (n), even if they were less than 5. I do not understand what "back-calculation of cells" means. I am not convinced that it is impossible to get these values, given that the total (N) in each category is known. Please clarify.

It is a condition of our ethics approvals that cell counts <5 are not reported, to reduce the risk of individuals being inadvertently re-identified. Refraining from reporting cell counts <5 is also recommended by NSW Health. Back-calculation refers to calculating the total within a cell based on other values (i.e. the denominator and the numerator in other categories). We have carefully endeavoured to ensure that the numbers in any cell cannot be back-calculated, including by suppressing additional cells within a row/column if necessary.

14. What does the overlapping of confidence intervals mean? (Lines 332-333). Based on this information, all–cause mortality was not different (or were similar) for the various months post-release in Figure 3. Please revise this paragraph.

We agree that the implications of this finding could have been more clearly stated, and have revised this paragraph as follows (lines 336 to 339):

Figure 3 depicts all-cause mortality rates per 1,000 person-years in different post-release time periods. All-cause mortality rate was highest in the first month post-release. However, given that the 95% confidence intervals for all mortality rate estimates overlapped, differences in the mortality rates for each time period cannot be conclusively inferred.

15. Strengths and limitations of the study should come after discussing the key findings, just before the conclusion.

We would normally structure the discussion as the reviewer suggests, however the BMJ Open Author Guidelines recommend discussion of strengths and limitations in the second paragraph of the discussion. We have therefore left this unchanged, but would be happy to move discussion of the strengths and limitations to the final paragraph on the advice of the BMJ Open editorial team.

16. Regarding selection bias, please clarify if selection bias originates from the nature of all the datasets available for use, or from the identification and selection process of the datasets used in the current study.

This is an inherent feature of the source datasets. We have added a sentence explicitly addressing this as follows (discussion lines 366 to 368):

A key limitation is representativeness and susceptibility to selection bias. This is an inherent limitation of the source datasets, which record service provision and are not a comprehensive registry of individuals with neurodevelopmental disabilities.

17. The modelling of time-varying covariates should be expressly mentioned in the methods first, before appearing in the discussion. Refer to my comment #10 above.

We specifically mention which variables in the Cox regression model are time updated, including the main exposure (as noted above), in the methods section when describing how these variables are ascertained.

However, we have also added mention of, and justification for, the time-updated nature of the health-related covariates in the 'statistical analysis' section as follows (lines 236 to 239):

Model 2 further adjusted for health-related covariates (co-occurring mental illness, drug-related harm, alcohol-related harm, CCI score), which we modelled as time-updated covariates to capture changing morbidity patterns given the youth of our cohort.

18. To make the title more concise, the phrase "neurodevelopmental disabilities" would suffice for "intellectual and other neurodevelopmental disabilities", even if most study participants had intellectual disability.

We have amended the title as suggested.

References

1. NSW Health Centre for Health Record Linkage. Quality assurance [Internet]. Centre for Health Record Linkage. n.d. [cited 2025 Aug 8]. Available from: https://www.cherel.org.au/quality-assurance

2. Spaulding AC, Seals RM, McCallum VA, Perez SD, Brzozowski AK, Steenland NK. Prisoner Survival Inside and Outside of the Institution: Implications for Health-Care Planning. Am J Epidemiol. 2011 Mar 1;173(5):479–87.

3. Kariminia A., Butler T.G., Corben S.P., Levy M.H., Grant L., Kaldor J.M., et al. Extreme cause-specific mortality in a cohort of adult prisoners - 1988 to 2002: a data-linkage study. Int J Epidemiol. 2007;36(2):310–6.

4. Vandenbroucke JP, Elm E von, Altman DG, Gøtzsche PC, Mulrow CD, Pocock SJ, et al. Strengthening the Reporting of Observational Studies in Epidemiology (STROBE): Explanation and Elaboration. PLOS Med. 2007 Oct 16;4(10):e297.

5. Trofimovs J, Dowse L, Srasuebkul P, Trollor JN. Using linked administrative data to determine the prevalence of intellectual disability in adult prison in New South Wales, Australia. J Intellect Disabil Res. 2021;65(6):589–600.

6. Hwang YI (Jane), Srasuebkul P, Foley KR, Arnold S, Trollor JN. Mortality and cause of death of Australians on the autism spectrum. Autism Res. 2019;12(5):806–15.

7. Reppermund S, Srasuebkul P, Heintze T, Reeve R, Dean K, Emerson E, et al. Cohort profile: a data linkage cohort to examine health service profiles of people with intellectual disability in New South Wales, Australia. BMJ Open. 2017 Apr 1;7(4):e015627.

VERSION 2 - REVIEW

Reviewer **2**

Name **Abuga, Jonathan**

Affiliation **Kenya Medical Research Institute Wellcome Trust Research Programme, Clinical Research (Neurosciences)**

Date **07-Sep-2025**

COI

All my comments and suggestions are adequately addressed
